# Prevalence and associated risk factors of hydatidosis in cattle slaughtered at Wolaita Sodo municipality abattoir

**DOI:** 10.1002/vms3.70008

**Published:** 2024-08-30

**Authors:** Habtamu Endale, Mesfin Mathewos

**Affiliations:** ^1^ School of Veterinary Medicine Wolaita Sodo University Wolaita Sodo Ethiopia; ^2^ School of Veterinary Medicine Wachemo University Wachemo Ethiopia

**Keywords:** abattoir, Ethiopia, hydatidosis, prevalence, Wolaita Sodo

## Abstract

**Background:**

Hydatidosis, caused by the larval stage of the tapeworm *Echinococcus granulosus*, affect cattle by forming hydatid cyst in thier lungs, livers and pose great financial loss in animal production and country's economy by both direct and indirect effect. Despite its great economic and health importance, there is an absence of current information on cystic echinococcosis in cattle slaughtered at Wolaita Sodo municipality abattoir in Ethiopia.

**Objectives:**

Current investigation determines the prevalence, organ distribution and fertility of hydatid cysts in cattle slaughtered at the Wolaita Sodo municipality abattoir in Ethiopia.

**Methods:**

A cross‐sectional study was conducted from February, 2023, to October, 2023, at Wolaita Sodo municipality abattoir in southern Ethiopia, through regular meat examinations and cyst characterisation to determine the prevalence, organ distribution and fertility of hydatid cysts.

**Results:**

The overall prevalence of hydatidosis recorede in current study was 17.9% (69/384) and has shown a statistically significant association (*p* < 0.05) with the body condition of cattle. However, there was no statistically significant association (*p* > 0.05) between the prevalence of hydatidosis and other risk factors such as sex, breed, production system and origin of animals. This study showed that the lungs and liver were the most affected organs in cattle, followed by the spleen and kidneys. On cyst characterisation, the majority of hydatid cysts were found sterile (55.4%) followed by fertile (38.8%) and calcified (8.7%) cysts. Out of 125 fertile hydatid cysts tested for survival, 18.8% (58/321) were viable and 20% (67/321) were nonviable.

**Conclusion:**

The finding of this study shows that cystic echinococcus was important health threat of the cattle and widespread in the internal organs of affected cattle causing significant economic loss by condemning edible organs that are not suitable for human consumption. Therefore, urgent and integreted preventive action is needed to disrupt the life cycle of cystic echinococcosis to tackle subsequent financial loss and risk of zoonosis to humans in the study area.

## Graphical abstract

As the finding of the study revealed, about 17.9% of the cattle subjected to this study were found infected by hydatidosis, and body condition of the animals showing significant association with the prevalence of the disease. The most affected organs were lung and liver, followed by the spleen and kidneys. Cyst characterisation revealed that 55.4% were sterile, 38.8% were fertile and 8.7% were calcified, with 18.8% of fertile cysts being viable.

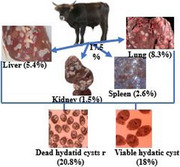

## 1 INTRODUCTION

Ethiopia is considered to have the largest livestock population in Africa which is estimated that the country is home to approximately 54 million cows, 25.5 million sheep, 24.06 million goats, 50.38 million poultry, 1 million camels and 5.21 million beehives (Asegede et al., 2015).

Livestock production in Ethiopia is an integral part of almost all farming systems in the highlands and the major occupation in the lowlands. Livestock in general and cattle in particular play a significant role as they are used for farm traction, farm fertilisation, and fuel (through manure) (Hassen et al., 2007). Despite the large population, livestock productivity remains low due to high livestock disease, malnutrition and management constraints (Kumsa, 2019). Among the many prevalent livestock diseases, parasitosis is a major health problem that impedes the productivity of livestock in tropical regions, including Ethiopia (Guduro & Desta, 2019).

Hydatidosis, also known as hydatid disease or Cystic echinococcosis, is one of the widespread chronic endemic helminthic diseases caused by the metacestode of the tapeworm *Echinococcus granulosus* (Al‐Khalidi et al., 2020), which affect the wellbeing and efficiency of cattle, sheep, goats, camels, buffaloes and pigs in several developing countries including Ethiopia (Kumsa, 2019; Romig et al., 2011).

Food animals (intermediate hosts) acquire infection by accidentally ingesting infectious eggs with contaminated grass or water, eggs later develop in the larval stage (metacestode) of parasites in many internal organs such as the liver, lungs, spleen, heart and kidneys, eventually causing pathological lesions associated with cystic echinococcosis (CE) (Singh et al., 2014). Humans can also be infected accidentally by ingesting infected eggs with contaminated water, vegetables, other foods, or in direct contact with dogs. The infection cycle is complete when the dog (definitive host) consumes offal with viable hydatid cysts (Budke et al., 2006). In contrast to the hydatid cysts of the intermediate host, adult tapeworms are harmless to dogs (Cardona & Carmena, 2013). Factors that determine the prevalence of echinococcosis are lack of proper meat management, poor livestock breeding, traditional backyard breeding practices, lack of sufficient awareness of food poisoning, and the presence of large stray dogs (Guduro & Desta, 2019).

Losses in productivity such as reduction in carcass weight, milk production, fleece and wool value, fertility, hide value, birth rate, and fecundity; delayed performance and growth; condemnation of organs, especially liver and lungs; and costs for the destruction of infected viscera are the major economic impacts associated with cystic echinococcosis in food‐producing animals (Singh et al., 2014). As a result, hydatidosis inflicts enormous economic damage in the sector and health and welfare threats in domestic ruminants in different parts of the country at different rate with limited or no preventive measures undertaken in Ethiopia (Melaku et al., 2012).

Hydatidosis in cattle has been conveyed from some parts of the country (Abebe et al., 2014; Kumsa, 1994; Mersie, 1993; Negash et al., 2013). Thus, wide‐ranging and up‐to‐date evidence is anticipated for the effective control and prevention of cystic echinococcosis in the country (Jobre et al., 1996; Magambo et al., 2006).

Despite its great economic and health importance, there is an absence of up‐to‐date information on cystic echinococcosis in cattle slaughtered at Wolaita Sodo municipality abattoir in Ethiopia. Thus, current investigation investigates the prevalence, organ distribution, and fertility of hydatid cysts in cattle slaughtered at the Wolaita Sodo municipality abattoir in Ethiopia.

## 2 MATERIALS AND METHODS

### 2.1 Study area

**FIGURE 1:**
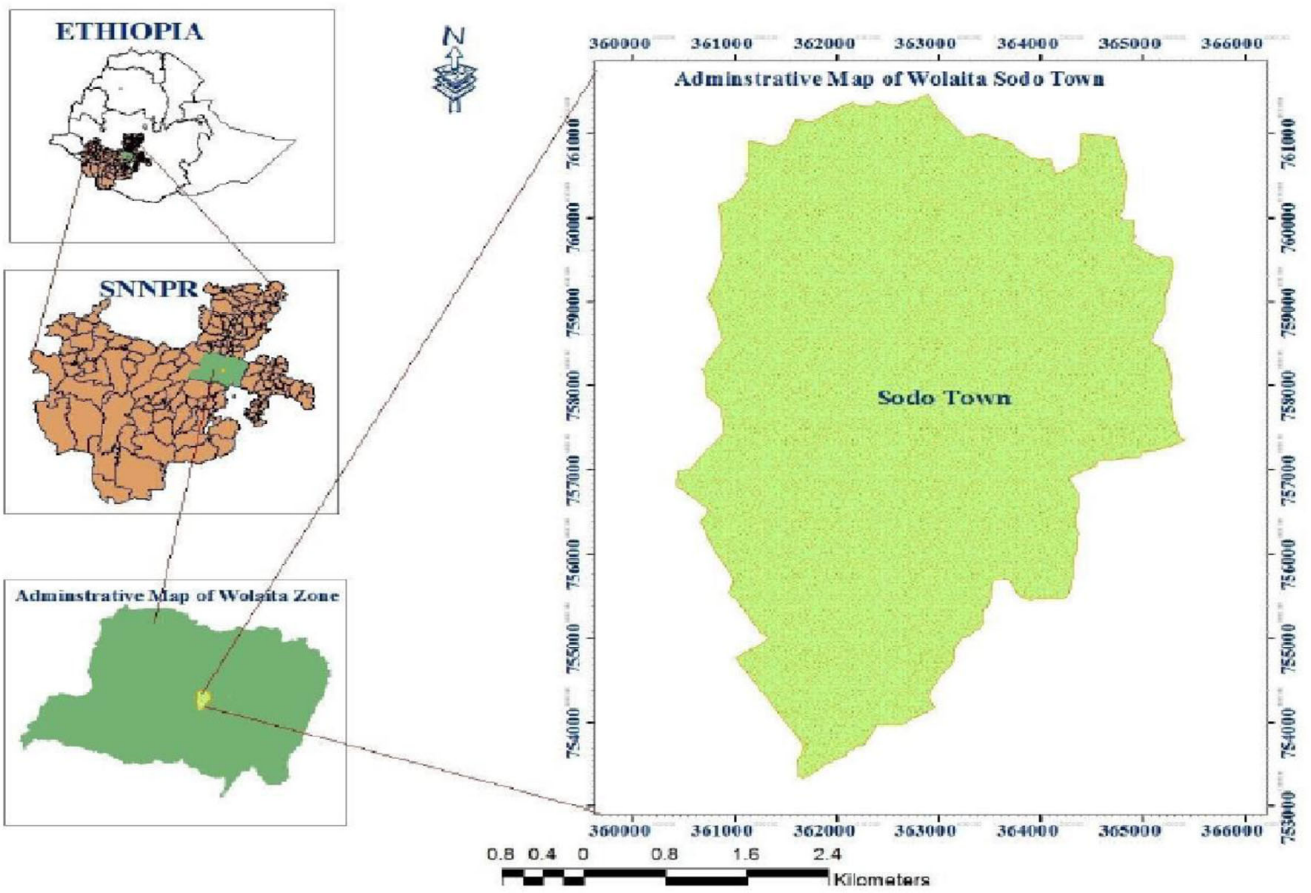
Map of the study area (Sodo town). Source: Elias (2021).

This study was conducted in  Wolaita Sodo municipality abattoir, which is located 390 km southwest of Addis Ababa following the tarmac road that passes through Shashamane to Arbaminch. Wolaita Sodo is a town of the Wolaita zone. It has a total area of 4541 km^2^ and is composed of 12 woredas and 3 registered towns (Figure 1). Livestock production in Wolaita zone includes cattle (oxen, milking cows and young stock), goats and sheep, equine (horses and equine) and poultry (CSA, 2014).

### 2.2 Study animals

The study animals included both male and female crossbred and native cattle, which were taken to the Wolaita Sodo municipal abattoir for slaughter. However, most of the animals slaughtered were Zebu cattle, and there were few hybrids.

### 2.3 Study design

A cross‐sectional study was conducted from February, 2023, to October, 2023, to determine the prevalence, organ distribution and characteristics of hydatid cysts in cattle slaughtered at the Wolaita Sodo municipality abattoir. For this purpose, three slaughtering‐day visits per week were made to the Wolaita Sodo municipality abattoir. All cattle slaughtered on each visit‐day were examined for hydatid cysts.

### 2.4 Sampling method

A simple random sampling technique was used in the cave to select the required number of study animals. Before sampling, each selected animal was given an identification number, and data on each animal's gender, age, breed and origin were recorded. During the meat examination, the identified animals and their respective organs were examined strictly and separately to avoid misperception between the organs. Meat inspections were performed according to the procedure of the Ethiopian Ministry of Agriculture Meat Inspection Ordinance (1972) for the detection of hydatid cysts. A visual inspection was performed, followed by several 0.5 cm incisions in each organ (Tolosa et al., 2009).

### 2.5 Sample size determination

A total of 384 cattle were examined to determine the presence of hydatid cysts by postmortem examination of different visceral organs (liver, lung, heart, kidney and spleen). This sample size was calculated as indicated by Thrusfield (2018), based on 50% expected prevalence and a 95% confidence interval with 5% desired absolute precision using the following formula:

$$N=\frac{Z^{2}\times P_{\exp}\left(1-P_{\exp}\right)}{d^{2}},$$

where *N* is required sample size, *P*_exp_ is expected prevalence, *d* is desired absolute precision.

### 2.6 Study method

#### 2.6.1 Ante mortem examination

Immediately before slaughter, a complete ante mortem examination survey of cattle was conducted. Cattle were examined for obvious signs of illness at rest and/or in gesture. All cows were tagged with an identification number before the commencement of slaughter, based on the markings listed on the body surface using ink when the cattle entered the stables. The age, sex, breed, production system and origin of each animal were recorded and divided into two groups as adults (7–9 years) and old (10+) as stated by (Herenda et al., 1994). Younger age groups are excluded from the study because most local farmers do not sell cattle when they are young, but most of the time after many years of traction. Moreover, slaughterhouses are not export slaughterhouses that are primarily focused on mature age. The age of each study cattle was estimated based on the dentition formula described by (Pace & Wakeman, 2003). Their body condition was categorized as good, medium and poor by adopting chriteria developed by Nicholson and Butterworth (1986). The origin of the animals was also obtained from the owners who brought the cows for slaughter.

#### 2.6.2 Postmortem inspection

Postmortem inspection was performed according to procedures recommended by food and agricultural organisations (Herenda et al., 1994). The internal organs, especially the lungs, liver, heart, spleen and kidneys, were carefully examined by visualisation, palpation and incision. Organs containing cysts from infected animals were collected and number of cysts were counted and recorded for each organ. The diameter of the collected cysts was measured and classified into small (less than 5 cm diameter), medium (5 cm to 10 cm diameter) and large (greater than 10 cm diameter) as described by Abebe et al. (2014), Kumsa (1994) and Negash et al. (2013). The fertility of each cyst was determined after reducing the pressure of the cyst fluid using a sterile hypodermic needle. The cyst was then incised with a sterile scalpel blade and the contents were poured into a glass Petri dish for inspection. The presence of protoscolices, which appear as white spots on the embryonic epithelium and attach to the germ layer in the form of a brood capsule or cystic fluid, was considered an indicator of fertility. Sterile cysts are characterised by their smooth inner layer, and the fluid they contain is usually slightly cloudy, but cysts identified as calcified make a gritty sound when incised. Fertile cysts were subjected to viability tests. Droplets of sediment containing protoscolices were placed on a microscope slide, covered with a cover glass, and an amoeba‐like peristaltic motion was observed with a 40× objective lens. For clear visibility, a drop of 0.1% aqueous eosin solution was added to an equal volume of precipitate containing protoscolices in the hydatid fluid on the microscopic slide (Negash et al., 2013). This technique distinguishes between dead (red) protoscolices and lives (colourless) protoscolices.

### 2.7 Data analysis

Data obtained from ant‐mortem (origin, sex, age, breed and body conditions) and post mortem findings was inserted in to Microsoft Excel 20016 spreadsheet computer program and filtered and cleaned. The data was analysed by using STATA for windows version 20 and a chi‐square test was applied to compare the prevalence of hydatid cysts among cattle of different sex, age, breed, origin and production system. A statistically significant association between variables is considered to exist if the *p*‐value is less than 0.05 at a 95 % confidence interval.

## 3 RESULTS

### 3.1 Prevalence of hydatidosis and associated risk factors

Of the total 384 cattle slaughtered and subjected to the study, 69 (17.9%) were determined to have one or more cysts in various internal organs (lungs, liver, spleen and kidneys). Among the putative risk factors, the occurrence of cystic echinococcus showed a statistically significant variation (*p* = 0.009) with the body condition of cattle. However, other factors has not shown a statistically significant difference (*p* > 0.05) with the existence of cystic echinococcus (Table 1).

**TABLE 1:** Prevalence of hydatidosis in cattle.

Factors	Number of examined	No. of infected	Prevalence (%)	*X* ^2^	*p*‐value
Origin					
High land	289	56	19.4	1.5721	0.210
Low land	95	13	13.6		
Production system					
Extensive	282	55	19.5	1.6966	0.193
Semi‐intensive	102	14	13.7		
Breed					
Local	349	62	17.78	0.1078	0.743
Cross	35	7	20		
Sex					
Male	274	52	18.9	0.6611	0.416
Female	110	17	15.4		
Age					
Adult	222	35	15.7	1.7326	0.188
Old	162	34	20.9		
Body condition					
Good	52	12	23.1	9.4895	0.009
Medium	254	35	13.7		
Poor	78	22	28.2		
Overall	384	69	17.9		

### 3.2 Hydatid cysts distribution in different organs

**FIGURE 2:**
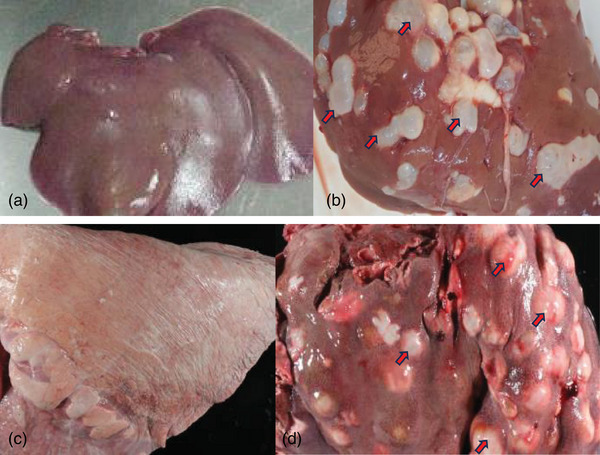
Hydatid cyst in different visceral organs. This figures illustrates hydatid cyst infected organs in comparison with normal ones. From above figures, (a) normal liver, (b) infested liver, (c) normal lung and (d) infested lung and red arrows dipict the hydatid cysts distributed in each organ.

**FIGURE 3:**
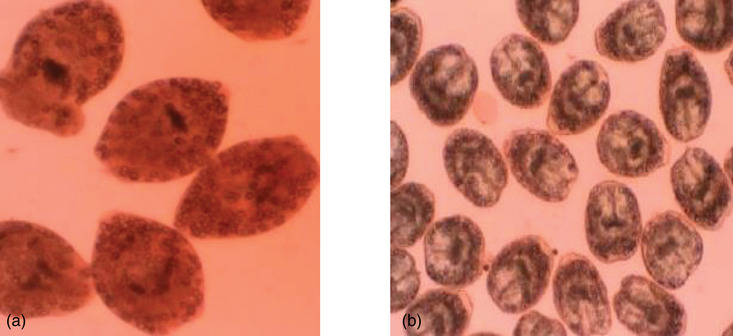
Microscopic image of the cycts viability. The dead hydatid cyct (a) is characterised by thickened and calcified fibrous wall, collapsed and degenerated germinal layer, inflammatory infiltrate within the cyst wall and calcifications within the cyst wall. Viable hydatid cysts (b) area characterised by intact and active germinal layer, clear or slightly turbid cycst fluid, containing daughter cysts (protoscolices) and hydatid sand (small particles), small invaginated protoscolexes and intact laminated membrane.

The study showed that the lungs (Figure 2d) and liver (Figure 2, 3) were the most affected organs in cattle, with the spleen and kidneys having infection rates of 8.3%, 5.4%, 2.6% and 1.5%, respectively (Table 2).

**TABLE 2:** Prevalence of hydatid cysts in different organs.

Organs	No. of affected organs	Percentage (%)
Lungs	32	8.3
Liver	21	5.4
Spleen	10	2.6
Kidneys	6	1.5
Total	69	17.9

### 3.3 Cyst characterization and  organ distribution

This study detected a total of 321 cysts from various organs with a prevalence of 48.3%, 33.3%, 15.6% and 2.8% in the lungs, liver, kidneys and spleen. Both fertile and nonfertile cysts were detected in cyst characterisation tests, except for the kidney, where only sterile cysts were detected. Of the total 125(38.9%) fertile cysts tested for survival, 18% (58/321) were viable (Figure 3a) and 20.8% (67/321) were nonviable cysts (Figure 3b). The majority of the hydatid cysts were sterile (55.4%) (Table 3).

**TABLE 3:** Characterisation of hydatid cysts in different organs of affected cattle.

	Fertile cysts		Nonfertile cysts		
Organs	Viable (%)	Nonviable (%)	Sterile (%)	Calcified (%)	Total (%)
Lungs	38 (24.5)	37 (23.8)	76 (49)	4 (2.5)	155 (48.3)
Liver	10 (9.3)	26 (24.2)	52 (48.5)	19 (17.7)	107 (33.3)
Spleen	0	4 (44.4)	5 (55.5)	0	9 (2.8)
Kidneys	0	0	45 (90)	5 (10)	50 (15.6)
Total	58 (18)	67 (20.8)	178 (55.4)	28 (8.7)	321 (100)

## 4 DISCUSSION

Hydatidosis is known as a major disease of livestock and humans in various parts of the world, and its prevalence and economic importance have been conveyed by different scholars in different parts of the world. In this study, the occurrence of hydatid cyst in cattle of various breeds, ages, sex, origin and production systems slaughtered at Wolaita Sodo municipality abattoir was consistent with the finding of Jobre et al. (1996), Kumsa (1994) and Kumsa (2019). The overall prevalence of hydatid cyst (17.9%) in cattle slaughtered in the study site was very much closer with earlier reports of Gebremeskel and Kalayou (2009), Kebede et al. (2009a) and Regassa et al. (2009) who reported a prevalence of 17.5%, 16% and 15.4%, respectively. However, it was lower than the reports from other parts of Ethiopia, such as 84.3% in Gondar, 68.9% in Injibara, 73.4% in Finoteselam by Kebede (2010), 61% in Asela by Koskei (1998), 52.69% in Hawassa by Regassa et al. (2009), 49.5% in cattle slaughtered at Shashemanne Municipal Abattoir in Oromia by Negash et al. (2013), 48.9% in Debre Markos by Kebede et al. (2009a), 46.5% in Debrezeit by Jobre et al. (1996), 34.05% in Bahirdar by Kebede et al. (2009b), 32.1% in Mekelle by Berhe (2009), 29.7% in Ambo by Zewdu et al. (2010), 22% in Tigray by Kebede et al. (2009b), 21% in Ababa abattoir enterprise by Kumsa (2019), and 20.5% in cattle slaughtered at Gondar Elfora Abattoir in northern Ethiopia by Abebe et al. (2014). In other studies from Ethiopia, hydatid cyst prevalence in cattle has been reported as lower than that in our study (8.5%) in Gofa by Regassa et al. (2009). This variation in the prevalence of cystic echinococcosis in cattle in different regions of Ethiopia may be ascribed to the differences in agroecology, the timing of studies, stock density and migration of animals, livestock production systems, awareness, culture, and religion of society and attitudes, and attitudes to dogs in different parts of the country (Abebe et al., 2014; Kumsa, 1994; Romig et al., 2011).

In this study, a statistically significant difference (*p* < 0.05) was seen between the body condition of cattle and the occurrence of cystic echinococcus. It was found that animals with poor body conditions had a large number of cyst counts, which was likely reflected the effects of cyst load in poor body conditioned animals. Polydorou (1981) states that when the infection is moderate to severe, parasites can cause delayed performance and growth, poor meat and milk quality, and weight loss. In the other hand, there were no statistically significant differences in origin, breed, gender or production system in the development of cystic echinococcus. The lack of significant differences in the prevalence of hydatid cysts in cattle of different breeds, agroecology and production systems might be due to a high population of stray dogs as well as wild dogs in close association with the family and farm animals in all agroecological zones of Ethiopia previously suggested by Abebe et al. (2014), Kumsa (2019) and Negash et al. (2013). Furthermore, the widespread outbreak of hydatid cysts in Ethiopia is attributed to the common practice of slaughter of ruminants in the backyard and roadside, a widespread tradition of providing uncooked infected offal to dogs and cats. Supported by several factors, including low public awareness, lack of proper fences and disposal pits from slaughterhouses that are easily accessible to dogs and other carnivorous animals, lack of habit of disposing of dead wildlife or domestic animals, and unburied leftovers creating favourable conditions and maintaining the life cycle of *E. granulosus* in stray dogs and wild carnivorous animals (Kebede et al., 2009a; Regassa et al., 2009; Zewdu et al., 2010).

There were no statistically significant changes in the development of cystic echinococcus and age of the cattle, but the prevalence was higher in adult animals as compared with old age groups. Similar reports were previously documented by Zewdu et al. (2010) and Regassa et al. (2009) from Ethiopia and by Adinehbeigi et al. (2013), Azlaf and Dakkak (2006) and Carmena and Cardona (2013) globally. This may be primarily attributed to the fact that older animals are exposed to *E. granulosus* eggs for longer periods, in addition to their weak immunity to fight against the infection (Cardona & Carmena, 2013; Himonas et al., 1987).

Regarding the organ distribution of cysts, this study showed that the lungs and liver were the predominantly affected organs with the prevalence of 8.3% and 5.4%, respectively. This outcome was in agreement with the reports of Bekele and Butako (2011), Jobre et al. (1996), Kebede et al. (2011), Kumsa (1994), Mersie (1993), Njoroge et al. (2002), Omer et al. (2010) and Zewdu et al. (2010). This is because the liver and lungs have the first large capillary region that this parasite wandering oncospheres encounters between the portal pathways before the involvement of any other peripheral organ (Kumsa & Mohammedzein, 2014). The higher prevalence in the lung might also be associated with the fact that cattle are slaughtered at an older age. At this point, the capillaries of the liver dilate and most cysts enter the lungs. In addition, hexacanthic embryos can enter the lymphatic system and be transported through the thoracic duct to the heart and lungs. In that case, the lung was infected before the liver as previously proposed by Cardona and Carmena (2013), Ibrahim (2010) and Taylor et al. (2007).

Examination of cyst fertility and survival revealed that approximately 55.4% were sterile, 38.8% were fertile, and 8.7% were calcified cysts. The higher prevalence of fertile hydatid cysts was recorded in the lungs as compared to the liver and all other organs. The higher proportion of fertile cysts, make the lungs more important than any other organ as a potential source of infection due to the soft consistency of lung tissue allowing cysts to develop more easily and promoting their fertility (Getaw et al., 2010).

A higher rate of calcification of cysts was found in the liver rather than in the lungs. This may be related to the abundant connective tissue response of relatively tall reticular endothelial cells and organs that enclose cysts within the fibrous wall up to 13 mm thicker than other organs (Abebe et al., 2014; Kumsa & Mohammedzein, 2014; Regassa et al., 2010). Current study generally suggests that the majority of bovine hydatid cysts are not infectious to the final host. This finding supports the previous discussion of several researchers who claimed that sheep play a greater role as an intermediate host for cystic echinococcosis than cattle in Ethiopia (Erbeto et al., 2010; Kumsa & Mohammedzein, 2014). However, the high prevalence of fertile hydatid cysts (38.9%) in cattle slaughtered at the Wolaita Sodo municipality abattoir show that cattle still have some potential source of infection to dogs and another final host of this parasite. This observation is consistent with previous results of (Kumsa, 1994). The fertility of cysts could be affected by differences in the strain of *E. granulosus* (Njoroge et al., 2002; Romig et al., 2011). Cysts can have different fertility rates depending on geographic location, host, location, size and cyst type (Ibrahim, 2010). In addition, the fertility of intermediate host cysts may also be genotype‐dependent, but unfortunately, no genotype studies are available for any host in Ethiopia. Because of the highest prevalence and fertility of pulmonary hydatid cysts than all the other organs in our current study and the practices of feeding uncooked lungs to dogs and cats in Ethiopia plays a major role of lungs in cystic echinococcosis than any other organ.

## 5 CONCLUSION AND RECOMMENDATIONS

The results reported here indicate that cystic echinococcosis is widespread in the area from where the cattle slaughtered at Wolaita Sodo municipal abattoir were raised. Observation of fertile cysts in the investigated organs instigates that cattle still plays a role in the life cycle of this serious zoonotic disease and is a potential risk of transmission to other intermediate hosts and the zoonotic effect on the population of the study area. Thus, Safe disposal of affected internal organs from the reach of domestic and wild carnivorous animals and educating the population about cystic echinococcosis in the area is needed to disrupt the life cycle of cystic echinococcosis from the meat processing plant to potential hosts in the area. Further detailed study including the possible risk factors for the occurrence of hydatidosis in the area supplying cattle for this abattoir is paramount to implement integrated control and preventive measures.

## AUTHOR CONTRIBUTIONS

All authors have made substantial contributions to the conception and design, sample collection, and acquisition of data, manuscript write‐up and interpretation of data. All authors read and approved the final manuscript.

## CONFLICT OF INTEREST STATEMENT

All authors listed in this manuscript declared that there was no competing interest in the submission and subsequent publication of this article.

## FUNDING INFORMATION

This work was not supported by any funding sources or institutions.

## CONSENT FOR PUBLICATION

All authors have seen and approved the final version of the manuscript being submitted. They warrant that the article is the author's original work, has not received prior publication and is not under consideration for publication elsewhere.

## ETHIC STATEMENT

Written ethical approval and consent for this study were obtained from the Wolaita Sodo University Research Ethics and Review Committee. In addition to this, oral consent was also obtained from the cattle owners before commencing antemortem and postmortem examinations by explaining the aim of the study verbally, by ensuring as the study will not cause any or little harm to their animals and carcass quality and are free to leave the study if they desire.

## Data Availability

The datasets used and analysed during the current study are available from the corresponding author upon reasonable request.
